# Evaluating Genomic and Clinical Risk Factors for Alzheimer’s Disease in Individuals with Hypertension

**DOI:** 10.3390/biomedicines13061508

**Published:** 2025-06-19

**Authors:** Elizabeth Kim, Kevin Zhang, Miski Abdi, Wei Tse Li, Ruomin Xin, Jessica Wang-Rodriguez, Weg M. Ongkeko

**Affiliations:** 1 Department of Otolaryngology-Head and Neck Surgery, University of California, La Jolla, San Diego, CA 92093, USAkez015@ucsd.edu (K.Z.); miskiabdi96@g.ucla.edu (M.A.); wqu60lht@gmail.com (W.T.L.);; 2 Research Service, VA San Diego Healthcare System, San Diego, CA 92161, USA; 3 School of Medicine, University of California San Francisco, San Francisco, CA 94143, USA; 4 Department of Pathology, University of California, La Jolla, San Diego, CA 92093, USA; jwrodriguez@ucsd.edu; 5 Pathology Service, VA San Diego Healthcare System, San Diego, CA 92161, USA

**Keywords:** Alzheimer’s disease, risk factors, hypertension, obesity, SNVs

## Abstract

**Background/Objectives:** Alzheimer’s disease (AD) is a progressive neurodegenerative condition whose growing prevalence has become an increasingly important public health concern as the population ages. The lack of a definitive cure elevates the importance of identifying risk factors that are crucial for prevention efforts. Hypertension (HTN) and obesity have emerged as two highly widespread, interrelated conditions that have independently been associated with AD risk. Despite extensive research into AD pathology, the impact of obesity in a hypertensive population is not well explored. This study aims to investigate how obesity and blood pressure control within a hypertensive population may interact with genomic risk and environmental factors to influence AD incidence. **Methods:** A retrospective cohort of matched AD and normal patients diagnosed with HTN and taking anti-HTN drugs (*n* = 1862) from the All of Us database was analyzed. In this hypertensive cohort, obesity was significantly associated with increased AD risk. Genome-wide association studies (GWASs) were conducted on hypertensive AD individuals (*n* = 1030) and identified six single nucleotide variants (SNVs) that were associated with AD development in this population. **Results:** Obesity and Area Deprivation Index, a measure of socioeconomic status, were significantly associated with elevated AD risk within the hypertensive cohort. GWAS analysis identified six SNVs significantly associated with AD development among the hypertensive cohort. **Conclusions:** Our findings suggest that among hypertensive individuals, comorbid obesity and the Area Deprivation Index confer greater AD risk. These results highlight the critical need for obesity prevention and management strategies as part of Alzheimer’s risk reduction efforts.

## 1. Introduction

Alzheimer’s disease (AD) is the most common cause of dementia, currently affecting over 55 million individuals worldwide, a figure expected to triple by 2050 as global populations age [1]. Despite recent advances in diagnostic imaging and targeted therapies, there is still no curative treatment, and symptomatic management continues to be the standard of care. As such, there is a crucial public health need to focus on the identification and modification of risk factors that may delay or even prevent the onset of Alzheimer’s disease.

Approximately 40% of dementia cases have the potential to be prevented through better control of risk factors including hypertension (HTN), obesity, diabetes, depression, smoking, and low educational attainment [2]. Among these, obesity and hypertension are of particular interest due to their role in promoting vascular dysfunction, systemic inflammation, and metabolic impairment, which have been increasingly recognized as a major contributor to cognitive deterioration [3,4]. As such, identifying controllable risk factors, such as HTN and obesity, has become a public health priority for delaying or preventing disease onset [2]. Previous studies have demonstrated that HTN is significantly associated with an increased risk of AD [5,6]. Mechanistically, elevated blood pressure may cause damage to the blood–brain barrier, promote cerebral microinfarcts, and induce white matter lesions which can collectively accelerate neurodegeneration [3,7]. Moreover, HTN may further compound cognitive risk due to its often co-occurrence with other metabolic comorbidities [3,5].

Obesity has emerged as a risk factor for dementia and cognitive impairment. In large population studies, obesity has been associated with an increased risk of late-life AD [8,9,10], mediated through mechanisms including chronic low-grade inflammation, insulin resistance, altered lipid metabolism, and adipokine dysregulation, all of which may promote the accumulation of amyloid plaques and accelerate neurodegenerative processes [11,12]. More importantly, HTN and obesity often coexist, creating complex metabolic syndromes. Specifically, studies have suggested that HTN, when combined with obesity may amplify the risk of cognitive dysfunction to a greater extent than either condition independently [13,14]. However, few studies have specifically focused on assessing whether comorbid obesity confers additional risk for AD development in hypertensive populations.

Socioeconomic factors, like the deprivation index, have been increasingly recognized as social determinants that influence cognitive health outcomes. The Area Deprivation Index (ADI) has shown the potential link between disadvantaged neighborhoods and poorer cognitive function, which aligns with previous smaller studies [15]. Furthermore, higher ADI scores, indicating greater deprivation levels, have been found to be associated with increased AD neuropathology and cognitive decline [16].

In this study, we leveraged the All of Us (AoU) Research Program, a large, nationally representative database, to extract a large electronic health record (EHR)-derived cohort of individuals with hypertension in order to evaluate the association between obesity and AD incidence within a hypertensive population. Using this resource, we identified relevant clinical variables. Refinements in dementia risk predictions have been made possible by advancements in genomic profiling. Genome-wide association studies (GWASs) created new opportunities to identify multiple loci implicated in AD. We integrated clinical predictors to assess their contribution to AD risk and compared AD-associated SNVs in the HTN cohort to those reported in the existing literature on the general population. By focusing our study on the hypertensive population, our study contributes new insights into aspects of AD pathogenesis that are not well understood and underscores the connection between metabolic health and AD development.

## 2. Materials and Methods

### 2.1. Data Extraction

Patient electronic health records (EHRs) were obtained from the All of Us (AoU) Controlled Tier Dataset v8. The data was filtered and compiled within the AoU dataset workspace. Dataframes containing patient information were downloaded using Python (v3.10.16) in the AoU workbench.

### 2.2. Data Preparation

Patient dataframes were merged and filtered to include only patients older than 40 years who were diagnosed with HTN and prescribed anti-HTN drugs utilizing Python. Individuals diagnosed with AD before the age of 40 were excluded. Patients with AD were matched to a non-AD patient based on age, race, and sex at birth. This study includes 931 AD patients and 931 non-AD patients. Covariate balance between the matched cohorts was assessed using density plots, histograms, and grouped bar plots to evaluate the effectiveness of matching.

Blood pressure control was defined as an SBP of less than 120 mmHg; an SBP equal to or greater than 120 mmHg was considered uncontrolled. Due to the multiple measurements of SBP for each patient, the average of the measurements taken within a year of taking HTN drugs was utilized as the SBP of an individual. Obesity levels were categorized into four classes based on BMI: Not Obese (<30), Class 1 Obesity (30–34.9), Class 2 Obesity (35–39.9), and Class 3 Obesity (≥40). To maintain consistency, due to multiple BMI measurements, the average of the measurements within a year of taking HTN drugs was used as the BMI of a patient. Implausible BMI measurements were excluded by retaining only those between 10.0 and 60.0.

A dataframe was created for only patients older than 40 years who were not diagnosed with HTN. Patients were matched based on age, race, and sex at birth. The non-HTN cohort includes 222 AD patients and 222 non-AD patients. SBP and BMI were calculated as the average measurement since the age of 40. The remaining risk factors were calculated using the same method for the HTN cohort.

### 2.3. Statistical Analysis

All analyses were conducted in Python using Pandas (v2.2.3), scikit-learn (v1.3.0), lifelines (v0.30.0), scipy (v1.11.2), matplotlib (v3.7.2), and seaborn (v0.12.2). A Choropleth map was created by utilizing the GeoPandas (v1.0.1) and GeoPlot (v0.5.1) Python libraries to plot the incidence per state in the U.S. The first three digits of patients’ zip codes were utilized to determine the state of AD incidence.

Kaplan–Meier survival curves for AD incidence were determined separately for each of the three cohorts: SBP control, obesity levels, and the combination of SBP control and obesity levels. Comparisons between groups were evaluated using the log-rank test. Patients missing follow-up time or AD status were excluded from all analyses. The follow-up time, measured in days, was the time after the age of 40 to either AD diagnosis or the current date for non-AD patients.

The multivariate Cox proportional hazards regression model was then employed to investigate the effects of several variables on the time to AD diagnosis. Blood pressure control and sex at birth were treated as binary categorical variables; race was one-hot encoded into multiple binary categorical variables, and BMI was categorized as an ordinal variable to track its progression. Hazard ratios (HRs) and the corresponding 95% confidence intervals (CIs) were computed to assess the association between covariates and time to AD diagnosis.

Pairwise comparisons between cohorts were performed. To visualize the significant associations (*p* < 0.05), forest plots were generated to display the HRs and CIs, with one group designated as the reference. Only cohort comparisons in which both groups had at least 30 individuals were included to reduce the risk of unreliable estimates due to small sample sizes.

Covariate distributions of years since birth, race, and sex at birth were examined across cohorts using density plots and bar plots to assess population differences.

### 2.4. Genome-Wide Association Study

Due to constraints within the All of Us data platform, we were unable to perform matched case–control selection. Therefore, we limited our analysis to individuals with Alzheimer’s disease, comparing those with hypertension to those without. This approach allowed us to leverage available data while controlling for AD status across all individuals. A total of 1030 variant call format (VCF) files of the short-read whole-genome sequencing (WGS) data were downloaded and extracted through the AoU Controlled Tier Dataset v8. These files consisted of 846 AD individuals diagnosed with HTN and 184 HTN-free AD individuals. Genotype data were obtained from the NIH Genomic Data Commons and accessed through the Terra platform. VCF files were processed utilizing Hail (v0.2) on a Google Cloud Dataproc cluster. Only individuals with a diagnosis of Alzheimer’s disease were included. Quality control was performed by removing samples with genotype call rates below 90% or mean genotype quality below 10. Only biallelic SNVs on autosomal chromosomes with call rates above 98%, minor allele frequency (MAF) greater than 1%, and Hardy–Weinberg equilibrium (HWE) *p*-values above 1 × 10^−6^ were retained. Genome-wide association testing was performed using logistic regression under an additive genetic model. The binary outcome was hypertension status, and the predictor was the number of alternate alleles at each SNV. Covariates were not included. Genome-wide significance was defined as *p* < 5 × 10^−8^.

## 3. Results

### 3.1. Study Demographics

**Figure 1:**
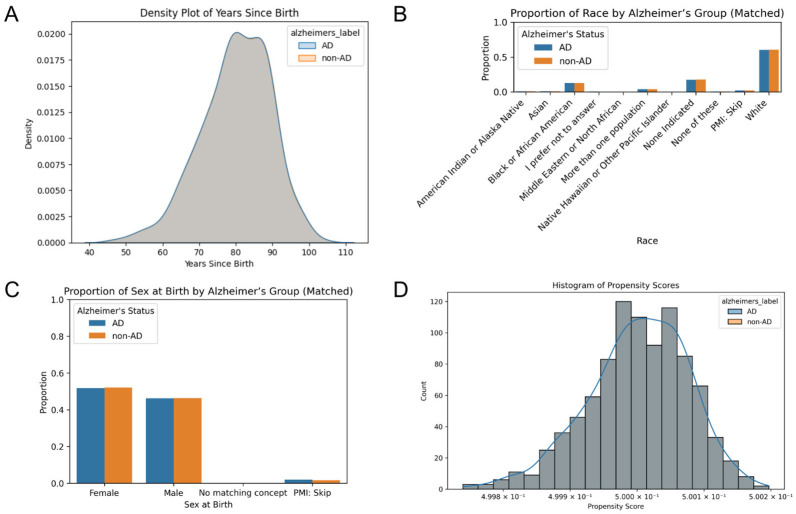
Comparison of covariation distributions between AD and control cohorts. (**A**) Density plot of years since birth illustrates the distributions between cohorts after matching. Grouped bar plots display the proportion of (**B**) race and (**C**) sex at birth across the matched cohorts. (**D**) A histogram of propensity scores that overlap indicates the balance of covariates.

The 931 AD individuals with hypertension were matched by age, race, and sex at birth to a non-AD patient. The density plot of years since birth shows the overlapping distributions between the two groups, indicating that there was a near-identical distribution between groups after matching (Figure 1A). Racial categories were balanced across the various groups, with similar proportion across the categories (Figure 1B). White participants represented over 50% of the sample. The proportion of sex at birth was well-balanced between groups (Figure 1C). Furthermore, the histogram of propensity scores, the predicted probability of being in each group given the covariates, demonstrated an overlapping distribution (Figure 1D). This suggests that for almost every AD patient, there is a control with similar covariates.

### 3.2. Geographical Distribution in AD Incidence

**Figure 2:**
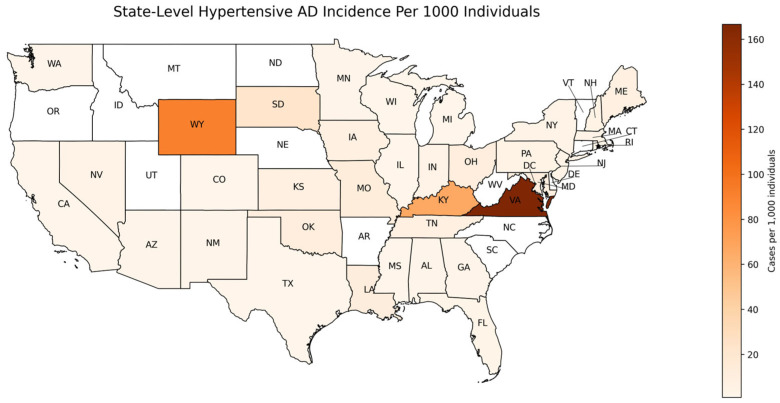
Geographic distribution of hypertensive Alzheimer’s disease incidence per 1000 individuals. Map showing the incidence of AD across different U.S. states. Regions shaded in darker colors represent higher HTN-predisposed AD incidence rates, while lighter colors represent lower incidence. The AD incidence was calculated for individuals with HTN who were prescribed anti-HTN medications and normalized to the total AoU cohort by state, regardless of HTN status. Incidence values are expressed per 1000 individuals.

To assess the regional variation in HTN-predisposed AD incidence, case distributions were mapped across the U.S. states (Figure 2). The geographical distribution was found as the proportion of individuals with both AD and HTN, normalized by the population of the overall AoU cohort in the same state regardless of HTN status. The choropleth map showcases the hypertensive AD incidence per 1000 individuals by state, the number of AD diagnoses out of every 1000 people in a given state. Virginia (VA), Kentucky (KY), and Wyoming (WY) exhibited the highest incidence of hypertensive AD after normalization, while states such as Oregon (OR), Vermont (VT), and South Carolina (SC) displayed lower incidence rates (Table S1). These findings highlight the geographical variability in AD incidence, with regions exhibiting differential disease occurrence.

### 3.3. Influence of Blood Pressure Control and Obesity on AD Incidence

**Figure 3:**
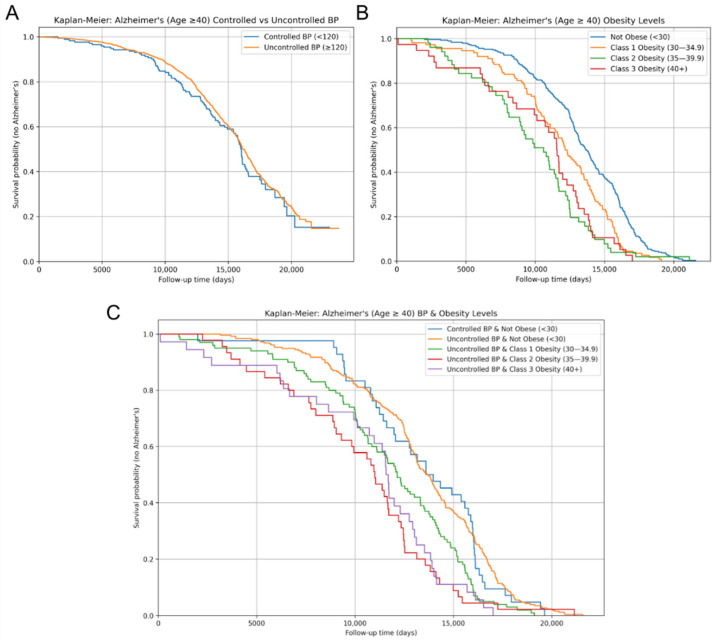
AD-free survival over time for risk factors. Kaplan–Meier survival curves for AD diagnosis of displaying AD-free survival over time between (**A**) controlled vs. uncontrolled systolic blood pressure; (**B**) obesity levels, categorized by BMI measurements; and (**C**) combined systolic blood pressure control and obesity levels, showing AD-free survival across joint categories. Survival probabilities indicate the proportion of individuals who remained AD-free over the follow-up period.

Kaplan–Meier survival analyses were employed to evaluate how SBP control and obesity level, both independently and jointly, affect the length of AD-free survival after the age of 40. The curves illustrate the cumulative AD incidence throughout time after the age of 40. Patients were stratified by SBP control status and obesity class. No significant difference in AD-free survival was observed between the uncontrolled and controlled SBP groups (log-rank *p*-value > 0.05) (Figure 3A). In contrast, obesity levels were significantly associated with differences in AD-free survival over time (Figure 3B). Patients that were classified as obese experienced the fastest decrease in the proportion of AD-free survival compared to the Not Obese patients. Class 3 Obesity exhibited the quickest decrease in AD-free survival proportion, followed by Class 2 and Class 1 Obesity, respectively. The log-rank tests revealed statistically significant differences between Not Obese and Class 2 Obesity (*p* = 0.0014), Not Obese and Class 3 Obesity (*p* = 6.61 × 10^−6^), and Class 1 and Class 3 Obesity (*p* = 0.0097). These findings suggest that certain obesity levels are associated with an increased risk of developing an earlier onset of AD compared to lower obesity or Not Obese groups.

AD-free survival curves stratified by both SBP control and obesity level revealed further significant differences in AD-free survival (Figure 3C). The Not Obese cohorts exhibited the slowest decrease in AD-free survival proportion, while the remaining three stratified groups followed similar survival trajectories. Among patients with uncontrolled SBP, AD-free survival was significantly shorter in individuals with Class 2 (*p* = 0.014) and Class 3 Obesity (*p* = 7.14 × 10^−6^) compared to the Not Obese group. Moreover, within the uncontrolled SBP group, the log-rank test determined a significant difference in AD-free survival between Class 1 and Class 3 Obesity (*p* = 0.011). The observed differences between Class 2 and 3 Obesity, as well as Class 1 and Class 3 Obesity, within the uncontrolled SBP cohort support a potential trend of shorter AD-free survival with increasing obesity severity. Furthermore, patients with controlled SBP and Not Obese demonstrated a significantly slower decrease in AD-free survival proportion compared to those with uncontrolled SBP and Class 2 (*p* = 0.061) or Class 3 Obesity (*p* = 0.00056).

### 3.4. AD Risk Using Hazard Ratios

**Figure 4:**
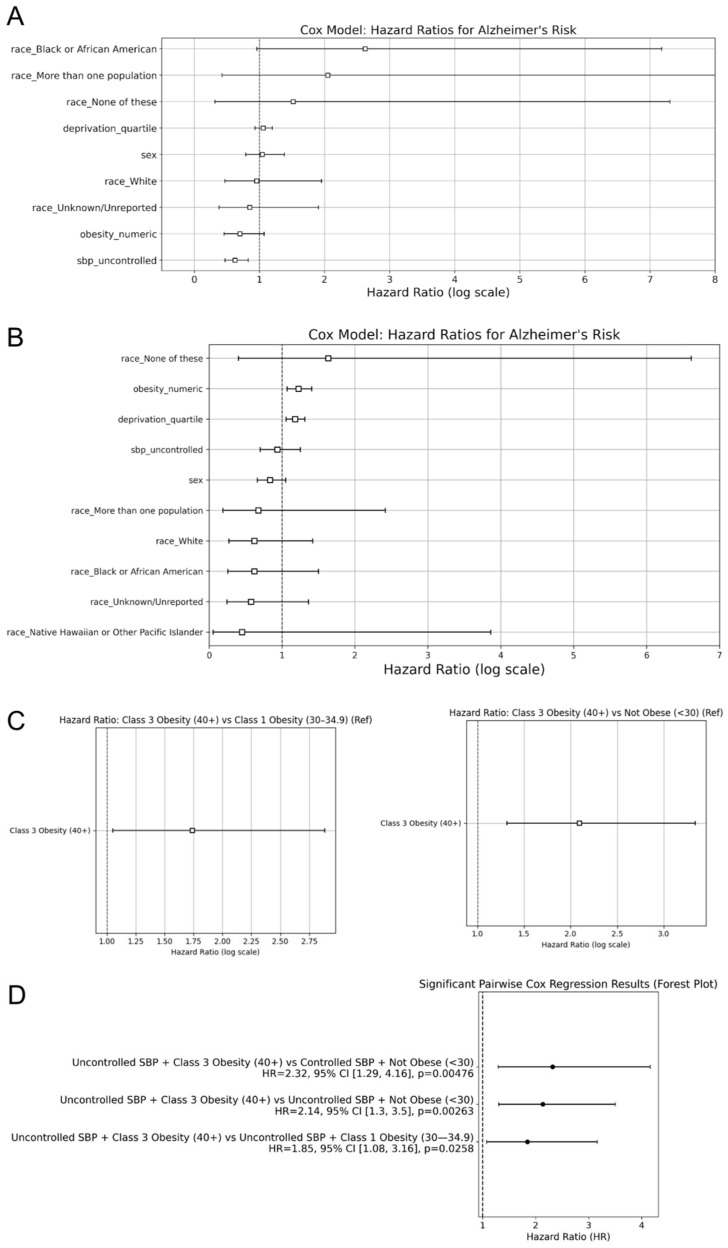
Hazard ratios for Alzheimer’s disease incidence. Hazard ratios, derived from the cox regression models, compare AD risk for (**A**) observed risk factors in the non-HTN cohort, (**B**) observed risk factors in the HTN cohort, and (**C**) obesity levels in the HTN cohort. A random forest plot displays the significant pairwise cox regression results from the (**D**) combination of systolic blood pressure control and obesity levels in the HTN cohort. The second factor for each comparison was considered the control.

To further quantify the contributions of risk factors to AD incidence, the Cox proportional hazards regression analysis was performed. After controlling for SBP, sex, race, and the deprivation index, in the non-HTN cohort, SBP control status emerged as the only significant predictor of AD risk (Figure 4A). Individuals with uncontrolled SBP had a 0.62-fold decrease in hazard of AD diagnosis compared to controlled SBP (*p* < 0.005). For the HTN cohort, obesity remained a predictor of AD (HR = 1.23, 95% CI: 1.07–1.41, and *p* < 0.005), while SBP control status was not statistically significant (*p* > 0.05; Figure 4B). Additionally, the deprivation index (HR = 1.18, 95% CI: 1.06–1.31, and *p* < 0.005) was significantly associated with an increased risk of AD. In AoU, the deprivation index is a zip code level-based measure of socioeconomic status, as it is based on education, income, poverty, housing, and health insurance coverage. The deprivation index was separated into quartiles, with quartile 1 representing individuals in the least deprived areas and quartile 4 representing the most deprived individuals.

In addition to the multivariate Cox analysis, pairwise Cox regression models were used to compare the AD risk of HTN individuals between specific groups from the Kaplan–Meier curves. These models revealed that individuals with Class 3 Obesity had a significantly elevated risk of AD. Individuals with Class 3 Obesity exhibited a significantly higher hazard of AD diagnosis compared to Not Obese individuals (HR = 2.10, *p* < 0.005; Figure 4C). Additionally, Class 3 Obesity had an increased hazard ratio compared to Class 1 Obesity (HR = 1.74, *p* = 0.03; Figure 4C). The remaining pairwise comparisons between obesity classes did not yield statistically significant results. These findings reinforce the Kaplan–Meier results and indicate that Class 3 Obesity confers the greatest risk of AD among the obesity categories examined.

To understand how SBP control interacts with obesity level in influencing AD incidence, additional pairwise Cox regression models were used for combinations of SBP status and obesity class for the HTN cohort. The significant results were then displayed on a forest plot to assess the effect size and significance across the different pairwise comparisons. Patients with uncontrolled SBP and Class 3 Obesity had a 2.14-fold increase in hazard of AD diagnosis compared to the uncontrolled SBP and Not Obese cohort (*p* < 0.005; Figure 4D). Similarly, compared to the controlled SBP and Not Obese cohort, uncontrolled SBP and Class 3 Obesity individuals had a 2.32-fold increase in hazards of AD diagnosis (*p* < 0.005; Figure 4D). Moreover, when SBP was uncontrolled, Class 3 Obesity had a significantly higher risk of AD compared to Class 1 Obesity (HR = 1.84, *p* = 0.026; Figure 4D). When SBP was controlled, there was no significantly different risk of AD between individuals with Class 1 and Class 3 Obesity (*p* > 0.05).

These findings suggest that obesity, particularly in higher classes, has a greater influence than SBP control on AD risk. SBP control did not significantly affect outcomes alone, but its interaction with obesity levels demonstrated different AD incidence rates, with Class 3 Obesity consistently exhibiting a greater risk of AD.

### 3.5. Genome-Wide Association Study (GWAS) of AD Risk

**Figure 5:**
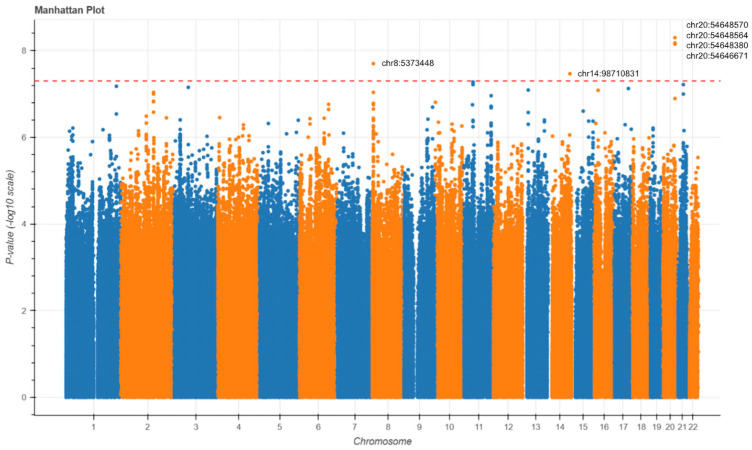
Manhattan plots of the GWAS for Alzheimer’s disease. GWAS Manhattan plots displaying SNVs associated with HTN in the full AD cohort. Each point has its chromosomal position on the x-axis and significance on the y-axis. Chromosomes have alternating colors for visual clarity. The dashed line indicates the genome-wide significance threshold.

To explore the potential genetic contributions to AD risk among HTN individuals, a GWAS was performed to compare AD patients with HTN to AD patients without HTN. Logistic regression was utilized to compare the genetic variant distribution between the two cohorts. The Manhattan plot revealed six loci with genome-wide significance (Figure 5). Notably, four different SNVs were identified at DOK5 on chromosome 20, and one SNV was identified at LINC02914 on chromosome 14. Additionally, an SNV on chromosome 8 is predicted by the UCSC genome browser to regulate PXMP2.

## 4. Discussion

In the context of global population aging, addressing the rising incidence of age-related diseases such as Alzheimer’s disease (AD) has become increasingly important. However, AD remains underexplored in hypertensive (HTN) populations, despite both conditions sharing common age-related risk factors and exhibiting similar trends in prevalence [17]. In addressing this gap of understanding AD, our study provides evidence that obesity and the Area Deprivation Index (ADI) are key predictors of AD risk among individuals within the HTN population. While prior studies have linked HTN, obesity, and the Area Deprivation Index individually to AD risk [4,5,6,8,9], our findings underscore the heightened vulnerability conferred by comorbid obesity and differential socioeconomic status within an already hypertensive population. Specifically, greater obesity and higher Area Deprivation Index values (higher levels of socioeconomic disadvantage) consistently displayed strong associations with earlier and more frequent AD diagnoses.

The relationship observed between increasing obesity class and AD incidence aligns with previous population-level studies linking obesity to late-life neurocognitive degeneration [8,9,10]. Our results extend these findings by demonstrating that obesity’s effect remains robust even within a hypertensive population. The interaction between obesity and SBP appears to biologically influence AD onset through a convergence of metabolic and vascular pathways. Central adiposity is associated with structural brain changes including atrophy and white matter degradation, as well as the disruption of blood–brain barrier (BBB) integrity, which is implicated in cognitive decline and increased AD risk. Adipose tissue is now recognized as an active endocrine organ that secretes adipokines, mediating inflammation, insulin resistance, and neurodegenerative processes. These adipokines may directly affect brain function by modulating microglial activation, neuroinflammation, and neuronal insulin signaling. Additionally, chronic obesity often leads to peripheral insulin resistance, which has been closely linked to central (brain) insulin resistance, a hallmark of early AD pathology. Proinflammatory cytokines released in response to amyloid-β accumulation phosphorylate IRS-1 at inhibitory serine residues, impairing insulin signaling and cognitive function. Elevated SBP exacerbates these effects by further compromising cerebrovascular integrity, reducing cerebral perfusion, and accelerating small vessel disease—all of which amplify amyloid deposition and tau pathology. These mechanisms support the observation that individuals with both obesity and hypertension experience a more pronounced relationship between beta-amyloid burden and cognitive decline, as shown in longitudinal neuropsychological studies [14]. Interestingly, this study reveals no significant association between SBP control alone and AD incidence. This may be due to the complexity of SBP’s temporal impact. Mid-life, rather than late-life SBP is more predictive of AD, or the variability in medication and treatment was not fully captured in our dataset. Individuals taking antihypertensive medications with previous damage due to longstanding hypertension prior to achieving SBP control are another limitation to control the ability of status to accurately reflect cumulative vascular burden. Additionally, this may be due to SBP thresholds that may not capture the full complexity of the range of vascular contributions to AD, especially when considering the variability of blood pressure readings. Furthermore, it is possible that metabolic disturbances associated with obesity exert a more direct or synergistic influence on neurodegeneration than SBP alone.

Furthermore, our findings align with a growing body of the literature suggesting that elevated neighborhood-level deprivation, measured by the Area Deprivation Index, contributes to heightened AD risk through multiple, interconnected pathways. AoU defines ADI as a composite metric based on education, health insurance, housing, income, and poverty. Individuals residing in disadvantaged neighborhoods are disproportionately exposed to adverse social determinants of health, including limited access to quality education, healthcare, nutritious food, and safe housing—all of which are associated with poor cognitive outcomes across the life course [15]. Notably, Powell et al. demonstrated that living in the most disadvantaged decile was associated with more than twice the odds of AD neuropathology at autopsy after adjusting for age, sex, and year of death [16]. Overall, these findings underscore the potential of ADI as a policy-relevant, currently overlooked AD risk factor and highlight the importance of incorporating socioeconomic status into disease management for AD.

Our genomic findings further enhance the interpretation of these results. The GWAS identified SNVs near genes such as DOK5, LINC02914, and PXMP2. DOK5 has previously been associated with reduced BMI and protection against obesity in a North Indian population, where specific variants were linked to a lower risk of obesity [18]. Given the observed association between obesity status and increased risk of AD in our study, these findings suggest that DOK5 may influence AD risk through metabolic pathways. PXMP2, peroxisomal membrane protein 2, was recently found to play a role in peroxisomal lipid metabolism [19]. LINC02914 has been less studied in the context of lipid metabolism, hypertension, or Alzheimer’s disease pathology, highlighting the need for additional functional studies to map its precise role in human diseases. We observed limited overlap between our GWAS signals and loci previously associated with blood pressure regulation, suggesting that the genetic architecture distinguishing hypertensive from non-hypertensive individuals within the AD population may be minimal. This raises the possibility that the shared pathways linking hypertension and AD are shaped more by environmental, lifestyle, or epigenetic factors than by distinct germline variations—consistent with our finding that the Area Deprivation Index is a significant risk factor for AD [20].

Contrary to previous studies, we found no statistically significant difference in hazard ratios between males and females after adjusting for age. These findings suggest that previously observed sex differences in AD incidence may be due to differences in life expectancy, with women living longer on average and thus having greater incidences of late-onset AD [21]. This is consistent with prior research showing that women tend to outlive men in nearly all populations worldwide, which leads to a widening sex gap in the oldest age groups, corresponding to a cohort where the risk of AD increases exponentially. Furthermore, by calculating the hazard ratio, which is time censored, instead of the odds ratio, which is calculated for an arbitrary time period, we obtained the instantaneous risk of developing AD instead of the cumulative risk over time. Given that AD diagnoses approximately double every five years beyond the age of 65, the overrepresentation of women in these oldest age brackets likely inflates female AD incidence statistics. Our results correct the widely held, oversimplified assumption that the female sex is intrinsically associated with greater AD susceptibility. Instead, our data support the interpretation that longevity, rather than sex-specific biology, is the cause of sex disparities in AD risk [22].

## 5. Conclusions

Our findings describe the multifactorial nature of Alzheimer’s disease risk within hypertensive populations, emphasizing the significant roles of metabolic dysregulation and socioeconomic disadvantage. Obesity and the Area Deprivation Index were significant predictors of AD risk, with genomic evidence suggesting dysregulated metabolic pathways as key mediators. The identification of loci such as DOK5 and PXMP2 suggests possible biological underpinnings that warrant further exploration, while the limited overlap with blood pressure-related loci highlights the importance of environmental and lifestyle factors in AD-HTN shared pathogenesis pathways. Additionally, the lack of sex-based differences in AD risk after adjusting for age calls for a re-evaluation of assumptions regarding females’ susceptibility to AD pathogenesis. These results underscore the urgency of adopting a more nuanced, integrative approach to AD risk stratification.

## Data Availability

The data presented in this study are available in the All of Us database (https://www.researchallofus.org/) (accessed on 7 May 2025).

## Supplementary Materials

The following supporting information can be downloaded at: https://www.mdpi.com/article/10.3390/biomedicines13061508/s1, Table S1: U.S. State Names to Two-Letter Abbreviations.

## Abbreviations

The following abbreviations are used in this manuscript:

AD	Alzheimer’s disease
HTN	Hypertension
SBP	Systolic blood pressure
GWAS	Gnome-wide association study
SNV	Single nucleotide variant
AoU	All of Us
EHR	Electronic health record
HR	Hazard ratio
VCF	Variant call format
WGS	Whole genome sequencing
MAF	Minor allele frequency
HWE	Hardy–Weinberg equilibrium
ADI	Area Deprivation Index
