# Supplementary material for: Evaluating Genomic and Clinical Risk Factors for Alzheimer’s Disease in Individuals with Hypertension
Source: Biomedicines. 2025 Jun 19;13(6):1508. doi: 10.3390/biomedicines13061508 (PMC12190856; doi:10.3390/biomedicines13061508)
Supplement: Supplementary file 1 [file biomedicines-13-01508-s001.zip › biomedicines-3661665-supplementary.pdf]

Supplementary Table S1. U.S. State Names to Two-Letter Abbreviations

U.S. State Names	U.S. State Abbreviations
Alabama	AL
Alaska	AK
Arizona	AZ
Arkansas	AR
California	CA
Colorado	CO
Connecticut	CT
Delaware	DE
Florida	FL
Georgia	GA
Hawaii	HI
Idaho	ID
Illinois	IL
Indiana	IN
Iowa	IA
Kansas	KS
Kentucky	KY
Louisiana	LA
Maine	ME
Maryland	MD
Massachusetts	MA
Michigan	MI
Minnesota	MN
Mississippi	MS
Missouri	MO
Montana	MT
Nebraska	NE
Nevada	NV
New Hampshire	NH
New Jersey	NJ
New Mexico	NM
New York	NY
North Carolina	NC
North Dakota	ND
Ohio	OH
Oklahoma	OK
Oregon	OR
Pennsylvania	PA
Rhode Island	RI
South Carolina	SC

South Dakota	SD
Tennessee	TN
Texas	TX
Utah	UT
Vermont	VT
Virginia	VA
Washington	WA
West Virginia	WV
Wisconsin	WI
Wyoming	WY
District of Colombia	DC
